# The Burden and Outcomes of Abdominal Pain among Children Presenting to an Emergency Department of a Tertiary Hospital in Tanzania: A Descriptive Cohort Study

**DOI:** 10.1155/2018/3982648

**Published:** 2018-05-09

**Authors:** Francis M. Sakita, Hendry R. Sawe, Victor Mwafongo, Juma A. Mfinanga, Michael S. Runyon, Brittany L. Murray

**Affiliations:** ^1^Emergency Medicine Department, Muhimbili University of Health and Allied Sciences, Dar es Salaam, Tanzania; ^2^Emergency Medicine Department, Muhimbili National Hospital, Dar es Salaam, Tanzania; ^3^Department of Emergency Medicine, Carolinas Medical Center, Charlotte, NC, USA; ^4^Division of Paediatric Emergency Medicine, Emory University School of Medicine, Atlanta, GA, USA

## Abstract

**Background:**

Abdominal pain in children can represent benign conditions or life-threatening emergencies. Aetiologies of paediatric abdominal pain vary geographically and have not been studied in acute care settings in East Africa. This study describes the clinical profiles and outcomes of children presenting with undifferentiated abdominal pain to the Emergency Department of Muhimbili National Hospital (ED-MNH).

**Methods:**

This was a prospective cohort study of children below 18 years of age presenting to the ED-MNH with abdominal pain. A structured case report form was used to collect data on patients from June to December 2016. Data included demographics, clinical presentation, and mortality. Data were summarised using descriptive statistics.

**Results:**

Out of 1855 children who presented to ED-MNH, 184 (9.9%) met inclusion criteria, and all were enrolled. The median age was 3.5 years (IQR: 1.3–7.0 years) and 124 (67.4%) were male. Most (138 [75.0%]) were referred from peripheral hospitals. The most frequent ED providers' diagnoses were hernia (34 [18.5%]) and intra-abdominal malignancy (19 [10.3%]). From the ED, 37 (20.1%) were discharged home, 83 (45.1%) were admitted to medical wards, and 48 (26.1%) were admitted to surgical wards. 16 (8.7%) underwent an operation. 24-hour, seven-day, and three-month mortality rates were 1.1%, 6.5%, and 14.5%, respectively. The overall in-hospital mortality rate was 12.2%. Multivariate analysis showed that age below 5 years, female sex, and haemoglobin less than 10.9 g/dl were significant factors associated with in-hospital mortality.

**Discussion and Conclusion:**

Abdominal pain is a common complaint among paediatric patients presenting to the ED-MNH. This presentation was associated with a high admission rate and a high mortality rate. Age below 5 years, female sex, and haemoglobin less than 10.9 g/dl were associated with mortality. Further studies and quality improvement efforts should focus on identifying aetiologies, risk stratification, and appropriate interventions to optimise patients outcomes.

## 1. Introduction

Abdominal pain is a common complaint among paediatric patients arriving at the EDs worldwide [1–6]. Abdominal pain may be a result of underlying traumatic or nontraumatic pathology. Nontraumatic abdominal pain is associated with both medical and surgical conditions and these can range from a benign, self-limiting condition such as constipation to a life-threatening emergency such as appendicitis [2]. Overall, the burden of abdominal pain among paediatric patients presenting to EDs varies across the world. Studies in high-income countries (HICs) have documented that 5–8.0% of ED presentations by children are due to abdominal pain [7–9].

The underlying aetiology of abdominal pain in children varies with age and geographical location worldwide. A study in Turkey reported that children below seven years of age were more likely to complain of abdominal pain when suffering from upper respiratory infections as compared to children above seven years of age who were more likely to have surgical conditions such as appendicitis [8]. On the contrary, in China, younger children were more likely to have surgical conditions, such as incarcerated hernia and intussusceptions, and surgical conditions were infrequent in school-aged children [9]. In Tanzania and its neighbouring countries, the age variation of children with undifferentiated abdominal pain presenting to the EDs is unknown as the majority of studies have investigated single aetiologies of abdominal pain [5, 10–13].

Children presenting to the EDs may have other symptoms and signs accompanied with abdominal pain, which require careful evaluation. A study in HIC on evaluating clinical outcomes in children with abdominal pain categorized the symptoms and signs to be originating from either abdominal or extra-abdominal processes [14, 15]. Abdominal symptoms and signs include decreased appetite, vomiting, and abdominal tenderness, while the extra-abdominal symptoms are fever, headache, sore throat, cough, and increased urinary frequency [15, 16].

In HICs, early recognition and treatment of children with abdominal pain are stressed, and mortality rates are less than 1.1% [17–19]. On the contrary, in Sub-Saharan Africa (SSA), there is limited data on the outcomes of children with undifferentiated abdominal pain presenting to the EDs. However, for a few specific conditions mortality rates have been reported and were found to vary with the underlying pathology. For example, studies done on appendicitis in children in Tanzania, Sudan, South Sudan, Kenya, and Nigeria showed no mortality [5, 6, 12, 13], while mortality rates due to intussusception alone in children in Tanzania have been found to range from 15 to 25% [10, 11].

To our knowledge, there is a dearth of information on the clinical characteristics of paediatric patients presenting to the EDs with a complaint of abdominal pain in our setting. We aimed to characterise the burden of undifferentiated abdominal pain, its aetiologies, and its outcomes among paediatric patients presenting to the tertiary hospital in Tanzania. The information derived from this study will help understand the magnitude of the problem and set a foundation on which other studies can be conducted.

## 2. Methods

### 2.1. Study Design

We conducted a descriptive prospective cohort study of paediatric patients older than one month and younger than 18 years presenting to the ED-MNH. Data was collected for 24 weeks from June to December 2016.

### 2.2. Study Setting

The study was conducted at the ED-MNH located in Dar es Salaam. MNH is a 1500-bed tertiary teaching hospital that serves as a national referral hospital [21]. The ED-MNH was opened in 2010, making it the first public full-capacity ED in the country. The department operates 24 hours a day and attends to 150–200 patients each day. Approximately one-fourth of the patient volume is children below 18 years [22, 23]. The department has special rooms dedicated to paediatric care and these rooms are under the supervision of emergency medicine specialists and master's trained nurses who oversee the care given by junior doctors and nurses.

### 2.3. Sample Size Calculation

We calculated that a sample of 184 subjects would allow us to characterise the mortality rate among our study population with a 95% confidence interval (CI) width of 5%. Our assumptions for this calculation were an estimated mortality of 12.3%, based on a combination of published and unpublished data (and personal communication with Dr. M. Charles, Head of Department of Paediatrics, MNH, personal quarterly data on mortality, March 2016 unreferenced), and a 10% rate of loss to follow-up.

### 2.4. Study Procedure

Consecutive paediatric patients presenting to the ED-MNH were screened and those who were found to be eligible were offered study enrolment. The principal investigator or a trained research assistant initially obtained verbal consent from the parent/guardian and assent from 8-year-old and older children. For patients who met inclusion criteria, the parent/guardian was given study information and provided signed informed consent. Demographics, clinical presentation, ED diagnoses, and outcomes of all enrolled patients were collected from the parent or guardian interview and review of the electronic medical record (Wellsoft Corporation, Version 11, Somerset, NJ, USA). The workup of the children with abdominal pain was at the physician's discretion. The tests included finger-stick blood glucose measurements, malaria rapid diagnostic tests, venous blood gases, complete blood counts, renal function tests, liver function tests, ultrasound, plain abdominal X-rays, chest X-rays, and abdominal CT scans. All children were followed up in the hospital (if admitted) and through direct mobile phone call to their parents or guardians to determine their outcomes at the time of disposition from the ED-MNH, as well as at 24 hours, seven days, and three months.

### 2.5. Participants

All patients between 1 month and 18 years of age presenting to the ED-MNH with abdominal pain (including abdominal distension and related complaints such as passing currant jelly stool, drawing up of legs, and inconsolability) and whose parent or guardian consented were enrolled in our study. Exclusion criteria applied to all children who sustained burn injury on the abdomen and children with acute abdominal pain who developed cardiac arrest in ED-MNH before being enrolled in the study. A research assistant screened every child who presented during a convenience sample of 12-hour shifts and enrolled all patients who met the inclusion criteria. No parents or guardians of children who met the inclusion criteria declined participation. There was no specific number of days or nights shifts allocated for data collection. However, data was collected on alternate days of the week for a period of 24 weeks. The research assistant phoned the parent or guardian to determine mortality at 24 hours, seven days, and 3 months. A child was described as lost to follow-up if the research assistant or the principal investigator failed to reach the parent or the guardian by phone after at least 3 attempts on different days of the week over two weeks.

### 2.6. Key Outcome Measures

The primary outcomes of interest were the proportion of children with abdominal pain who presented to the ED-MNH, diagnoses, and disposition from ED (home, to medical and surgical wards, or to operating theatre). The secondary outcome was mortality, which we studied at 24 hours, seven days, and three months.

### 2.7. Data Analysis

Data were entered into Microsoft Excel (2007, Microsoft Corporation, Redmond, WA, USA) and analysed with StatsDirect version 3.0.133 (StatsDirect Ltd., Cheshire, UK). Descriptive statistics (counts, percentages, medians, interquartile ranges, and 95% confidence intervals [CIs]) were generated for demographic characteristics, clinical features, and outcomes of patients in our cohort.

## 3. Results

**Figure 1:**
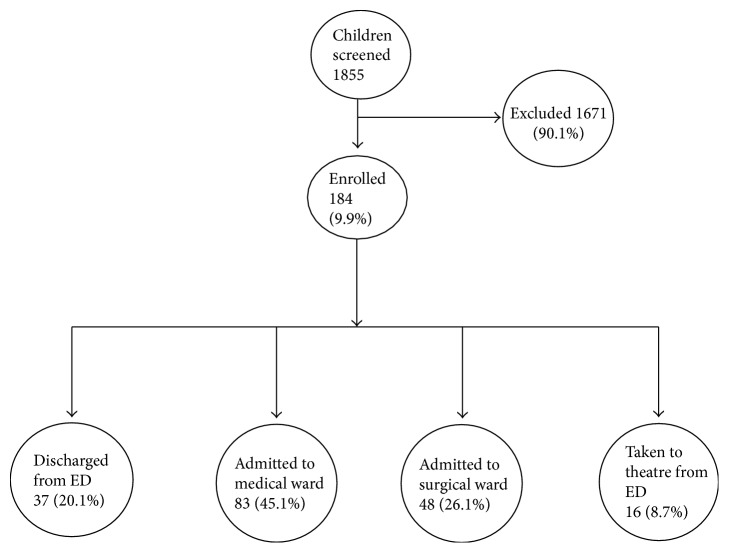
Screening and disposition of children presenting to the ED-MNH with abdominal pain.

We screened 1855 children below 18 years of age during the study period; 184 (9.9%) children were eligible for inclusion and parents of all consented to participate in the study. Among the 184 children, 37 (20.1%) were discharged home from the ED, 83 (45.1%) were admitted to the medical ward, 48 (26.1%) were admitted to the surgical ward, and 16 (8.7%) were taken directly to the operating theatre (Figure 1).

### 3.1. Demographic Characteristics

Of the 184 children enrolled, 124 (67.4%) were male and median age was 3.5 years (IQR: 1.3–7.0 years). The presenting episode of abdominal pain was the first episode for 111 (60.3%). The median duration of current illness was 4 days (IQR: 2.0–8.0 days). Overall, 138 (75.0%) children were referred from peripheral hospitals. Sickle cell disease was the most common comorbid condition, found in 17 (9.2%). Demographic characteristics are shown in Table 1.

After a careful history and physical examination, the most common reported associated symptoms were fever (93 [50.5%]) and vomiting (77 [41.8%]). An elevated temperature was present in the ED in 33 (18.1%) children and 14 (7.6%) had increased respiratory rate. None of the children had hypoxia (oxygen saturation < 95%) or bradycardia. The most common abdominal findings identified by the providers were distension and obvious swelling/mass in 95 (51.6%) and 55 (29.9%) children, respectively (Table 2).

The most frequent ED diagnoses among children below and above 5 years of age are shown in Table 3.

None of the children died in the ED. Hospital mortality was 12.2%. Disposition and mortality are shown in Table 4.

Overall, 16 (8.7%) children were taken to operation room, while 168 (91.3%) were managed conservatively; the in-hospital mortality rates in these groups were 3 (18.8%) and 15 (8.9%), respectively.

## 4. Discussion

In our cohort, 9.9% of paediatric patients who presented to the ED-MNH had a complaint of abdominal pain, similar to the reported rates of up to 8.1% from HICs [7, 9]. To the best of our knowledge, this is the first study to document the burden of abdominal pain among paediatric patients presenting to the emergency department in East Africa.

Three-quarters of children with abdominal pain were referred from other peripheral hospitals; this is a similar proportion to what is observed in other paeditaric patients presenting to ED-MNH [22]. The existence of a referral system in Tanzania requires a patient to be evaluated and managed by a lower-level health facility before being transferred to a higher, more advanced facility with experts in different fields for further management [24, 25]. The nature of the referral system may result in higher acuity in the patients that are seen in our ED and may lead to children being seen in advanced stages of their illness, contrary to what is observed in most of HICs.

The most frequently reported symptoms associated with abdominal pain were fever and vomiting. The findings are similar to studies in HICs, where these symptoms were often observed in undifferentiated cases of abdominal pain [8, 16]. However, in terms of diagnostic value, these most commonly reported symptoms have limited utility as they could be due to both medical and surgical pathologies found in our cohort.

After ED evaluation, the most frequent specific ED diagnoses in children below 5 years of age were different from those of individuals above 5 years of age. Moreover, this was in contrast to similar studies from other countries in Sub-Saharan Africa. Specifically, it does not align with a study in Central African Republic, where Séréngbé et al. found that appendicitis was the most common diagnosis found in children aged 2–10 years presenting with acute abdominal pain in a paediatric hospital [26]. This does not necessarily correlate with the most common symptoms of fever and vomiting that were found in our patients. We believe that because the diagnoses of hernia and intra-abdominal malignancies have specific examination findings, they are more likely to be specifically diagnosed in the ED. Limited availability of CT scans may result in poor diagnostic specificity in these patients. Furthermore, we believe that the patient population in our study may have been largely influenced by the nature of the referrals MNH receives due to the presence of specialised paediatric surgical and oncologic services [25].

Three-quarters of children with abdominal pain were admitted after evaluation at the ED. This highlights the high acuity of illness among children in our cohort. This high acuity is also reflected in the in-hospital mortality rate of this cohort. More than 12% of children with abdominal pain died in the hospital. This is higher than the 8% mortality rate reported in a previous study in the Central African Republic [26]. In HICs, the mortality rate is much lower, about 1.1% among the children who were hospitalised due to various aetiologies of abdominal pain [17–19].

Overall, our study showed that abdominal pain is a common presentation among paediatric patients presenting to the ED-MNH and that these children have a high mortality rate. The disparity between the results in our setting and those in other countries highlights the need for further research and quality improvement efforts focusing on diagnostic accuracy, risk stratification, and appropriate interventions to optimise outcomes of patients presenting to the ED with abdominal pain.

## Figures and Tables

**Table 1:** Demographic characteristics of children presenting with abdominal pain.

Variable	Overall
	*N* = 184
	*n* (%)
Male	124 (64.7)
Age in years, median (IQR)	3.5 (1.3–7.0)
Below 5 years	114 (61.9)
Above 5 years	70 (38.1)
First time abdominal pain	111 (60.3)
Duration of current illness (days), median (IQR)	4 (2.0–8.0)
Time to presentation to any hospital, median (IQR)	2 (1.0–6.0)
Referred from peripheral hospitals	138 (75.0)

**Table 2:** Reported associated complaints, vital signs, and physical findings.

Variable	Overall
	*n*/*N* (%)
*Reported associated symptoms*	
Fever	93/184 (50.5)
Vomiting	77/184 (41.8)
Diarrhoea	25/184 (13.6)
Cough	37/184 (20.1)
Decreased appetite	17/184 (9.2)
Weight loss	9/184 (4.9)
Nausea	5/184 (2.7)
*Vitals signs*	
Temperature > 37.5°C	33/182 (18.1)^*∗*^
Tachypnoea^*∗∗*^	14/184 (7.6)
Tachycardia^*∗∗*^	18/184 (9.8)
Capillary refill > 2 seconds	6/184 (3.3)
AVPU (abnormal)	9/184 (4.9)
*Abdominal findings*	
Distension	95/184 (51.6)
Obvious mass/swelling	55/184 (29.9)
Tenderness	55/184 (29.9)
Normal Examination	33/184 (17.9)
Decreased bowel sounds	12/184 (6.5)
Increased bowel sounds	11/184 (6.0)

AVPU: Alert, Verbal, Pain, Unresponsive. ^*∗*^2 children did not have temperature taken. ^*∗∗*^Based on vital signs according to age [20].

**Table 3:** Most common ED provider's diagnoses.

Age < 5 years	Overall (%)	Age ≥ 5 years	Overall (%)
Diagnosis	*N* = 114	Diagnosis	*N* = 70
Hernia (with/without obstruction)	31 (27.2%)	Sickle cell disease	11 (15.7%)
Intestinal obstruction	10 (8.8%)	Intra-abdominal malignancy	10 (14.3%)
Intra-abdominal malignancy	9 (7.9%)	Viral intestinal infections	8 (11.4%)
Abdominal pain of unknown origin	9 (7.9%)	Malaria	8 (11.4%)
Intussusception	7 (6.1%)	Appendicitis	5 (7.1%)
Malaria	7 (6.1%)	Abdominal trauma	4 (5.7%)
Sickle cell disease	6 (5.5%)	Constipation	4 (5.7%)
Lymphoma	5 (4.4%)	Hernia (with/without obstruction)	3 (4.3%)
Constipation	5 (4.4%)	Gastritis	3 (4.3%)
Hirschsprung	3 (2.6%)	Intestinal obstruction	2 (2.9%)

**Table 4:** ED disposition and mortality.

Variable	Overall	Confidence interval
	*n*/*N* (%)	
Discharged from ED	37/184 (20.1)	14.6–25.9%
Taken to theatre from ED	16/184 (8.7)	4.6–12.8%
Admitted to the hospital	131/184 (71.2)	64.5–77.4%
Died at the ED	0	
Mortality at 24 hours	2/184 (1.1)	−0.4–2.6%
Mortality at 7 days	12/184 (6.5)	2.9–10.0%
Mortality at 3 months	25/173 (14.5)^*∗*^	9.3–19.8%
Overall in hospital mortality	18/184 (9.8)	5.5–14.1%

^*∗*^11 children were lost to follow-up.

## Data Availability

The dataset supporting the conclusions of this article is available from the authors on request.

## Additional Points

*Limitations*. Our major limitation was the fact that this was a single-site descriptive study over a relatively short duration, limiting our account for seasonal variation of the abdominal complaints. Hence our results may not necessarily be generalizable to other EDs with different patient populations in different seasons.
